# The Influencing Mechanism of Interaction Quality of UGC on Consumers’ Purchase Intention – An Empirical Analysis

**DOI:** 10.3389/fpsyg.2021.697382

**Published:** 2021-07-19

**Authors:** Ruoshi Geng, Jun Chen

**Affiliations:** ^1^School of Management, Shanghai University, Shanghai, China; ^2^School of Continuing Education, Shanghai Jiao Tong University, Shanghai, China

**Keywords:** user-generated content, purchase intention, usefulness, trust, product involvement

## Abstract

User-generated content (UGC) refers to online information created and shared by consumers that can influence other users’ purchase decisions. Due to the rapid development of new technologies in business, marketers’ dependency on UGC is rising. This study aims to explain how the interaction quality of UGC affects its credibility as a source and the usefulness of its information, as well as how it influences consumers’ online purchase intention. In addition, we examine the moderating effect of product involvement. We collected data from 272 users of social media in China to conduct empirical research. The SPSS and Amos were used to analyze the data and test the hypotheses. The results reveal that UGC interaction quality is positively related to purchase intention, and this relationship is mediated by perceived usefulness and trust. Further, consumers’ product involvement negatively moderates the effect of perceived usefulness and trust on purchase intention. Findings from this study are expected to extend the literature on UGC and give benefits to marketers and online business operators.

## Introduction

With the emergence of web 2.0, the consumer market began a new round of consumption upgrading, and the structure changed from subsistence to enjoyment and development. Modern consumers get the purchase path through the search engine. They no longer passively accept information from businesses, but actively consult information created by other consumers (pictures, text, and video, etc.) as a reference for purchasing decisions (Thoumrungroje, 2014). User-generated content (UGC), a new network of information creation and organization, was born. UGC generally refers to the text, pictures, video, and other content created by users in any form published on the network. This includes purchase experiences shared by users on a website or app, a manifestation of the empowerment and decentralization of new media (Vickery and Wunsch-Vincent, 2007).

Alongside these changes, many e-commerce platforms are moving away from the traditional model of search and retail, and making use of pull strategies through content marketing to build relationships with customers. Content marketing is a relatively new technique used to create and distribute valuable or relevant content to acquire a defined target with the intention of obtaining profitable customer action (Steimle, 2014). Therefore, brands must recognize the importance of content marketing, understand the factors leading to its success, and the consequences of its application.

Despite increasing attention on content as a modern marketing tool, there are relatively few studies on the impact of UGC on consumers’ online purchase intention with an interactivity perspective (Müeller and Christandl, 2019). A key feature of social e-commerce is UGC, the product of interaction between users. For example, users can share shopping information on the platform, and other users can comment and exchange in a two-way flow of information. The networks in social media and positive interaction between users are the real source of content marketing. Therefore, it is crucial to refine the interactivity of UGC and explore its impact on consumers’ purchase intention.

This study explores the impact of UGC interaction quality on consumers’ purchase intention from the perspective of interaction. According to social cognitive theory, perceived interactivity, as an environmental factor, affects users’ behavior through human cognition (Cyr et al., 2009; Lee et al., 2011). To analyze the cognitive human factors, this study refines the mediating variables (perceived usefulness and perceived trust) based on the value theory. At the same time, for the information with the same content expression and dissemination direction, receivers’ understanding may differ. Therefore, we should consider the characteristics of information receivers when analyzing the impact of UGC on consumers. Product involvement in the communication effect has helped explain consumer behavior in advertising, promotion stimulation, information search, and processing (Zaichkowsky, 1985; Belvedere and Goodwin, 2017). Therefore, this study introduces consumer product involvement as the moderating variable.

This study contributes to the literature in three ways. First, based on the theory of social cognition, this study views interactivity as a key feature of Web 2.0 technology. It is an entry point to investigate consumers’ understanding of UGC interaction, highlighting the limitations of previous studies that only consider information characteristics and the role of individual social factors. We can then provide a basis for the research of perceived interactivity in the context of social media. Second, based on the value theory and the coexistence of media and social media platforms, this study views perceived usefulness and trust as mediating variables. This enriches the theoretical results of UGC behavior research on social media platforms. Third, by studying the impact of UGC on purchase intentions in social e-commerce, this study introduces product involvement as a moderating variable and provides a theoretical basis for social e-commerce to better play the role of UGC.

## Literature and Hypotheses

### Interaction Quality of UGC and Perceived Usefulness

The development of Web 2.0 technology created many UGC platforms. This made user interaction and the exchange of information possible, thus providing a broad choice for social media marketing (Deng et al., 2015; Felix et al., 2016). UGC is diverse and has strong media properties. To improve interaction quality, community members discard single text discussion and utilize more advanced forms of audio, video, pictures, and other resources (Zhao et al., 2011). Communication between members is more intuitive, the information richer and more reliable, and emotion more straightforward. The interactive quality of UGC emphasizes that the content generation process is also a good source of interpersonal interaction. This process includes not only interaction and feedback between consumers, but also a partnership between consumers and retailers.

Perceived usefulness is defined as the degree to which a person believes that the use of a system would improve one’s performance (Davis, 1989; Karahanna and Straub, 1999; Muslim et al., 2014). In this study, perceived usefulness is the overall wealth of usefulness in the message or information shared on social media. The virtual, anonymous, open, and weak group relationship among consumers means we cannot rely on identity recognition and acquaintance introduction to establish perceived usefulness. Only through online interaction can we obtain sufficient product information resources (Wu, 2007). The interaction of UGC effectively delivers information through engaging users’ attention, increasing their involvement, and enriching their experiences (Lee et al., 2011). The positive perception of interaction shows users they can update information quickly, in real-time, and actively control information acquisition on the platform (Animesh et al., 2011). Interaction between users is conducive to information collection and transmission, and helps other members gain opportunity benefits or key resources (Felix et al., 2016).

In the Internet environment, consumers can not only view product information, but browse the generated content of others. They can directly interact with others to obtain more comprehensive information and form their own useful perception of the product. Therefore, this study proposes hypothesis 1:

*H1:* Interaction quality of UGC is positively related to consumers’ perceived usefulness of products.

### Interaction Quality of UGC and Perceived Trust

High-quality interactivity can be the foundation of trust (Wu and Chang, 2005). This study defines trust as the willingness of consumers to be affected by integrity (honesty and commitment), kindness (caring, thinking for others), and skills (ability to meet requirements) in product recommendation based on UGC publishers. Communication and information exchange are key aspects of interactivity and have a significant influence on trust (Selnes, 1998). In social media, the relationship between users is a weak bond. Through continuous online interaction they can enhance their understanding, and then generate a sense of trust (Blanchard et al., 2011; Wang and Chen, 2012).

Effective interaction of UGC is helpful to find groups with common attributes among members of a virtual community. In groups with the same values or interests, it is easier to establish emotional ties, and ultimately enhance perceived trust (Müeller and Christandl, 2019). In addition, UGC interaction enhances information credibility. Consumers obtain information through UGC to reduce perceived risk and improve perceived trust (Li et al., 2020). The interactivity of social e-commerce platforms forms the basis of online word-of-mouth communication. Many consumers follow the trend of mass consumption. If consumers see many product comments and more members participate and share information, they will have a positive attitude toward the product or service (Ye et al., 2011). Once consumers frequently participate in UGC, they will continue to increase their understanding of UGC and to reduce the perceived risk, which is conducive to consumers’ trust in UGC (Dawes and Nenycz-Thiel, 2014). Therefore, this study proposes hypothesis 2:

*H2:* Interaction quality of UGC is positively related to consumers’ perceived trust of products.

### Perceived Usefulness and Purchase Intention

Perceived usefulness may affect online consumers’ response to UGC, influence their attitude, and lead to a purchase (Kim and Song, 2010; Muslim et al., 2014; Ventre and Kolbe, 2020). UGC adoption is a process where people use information purposefully (Pitta and Fowler, 2013). Consumers today usually use social media to find product information and customer feedback before a purchase decision as they are more dependent on UGC (Horst et al., 2007; Racherla and Friske, 2012; Dhahak and Huseynov, 2020). Consumers’ brand choice and purchase chiefly come from useful information. For UGC, all information that can help consumers make good purchase decisions will have certain use value, especially with unfamiliar product price and channel quality (Featherman and Pavlou, 2002; Racherla and Friske, 2012). It even exceeds demand for product price. This is because previous consumers usually have experience with the products they are planning to buy, which helps potential buyers make purchase decisions. Sharing information can help consumers reduce their perceived risk in decision-making (Horst et al., 2007; Racherla and Friske, 2012; Muslim et al., 2014). Therefore, this study proposes hypothesis 3:

*H3:* Perceived usefulness is positively related to consumers’ purchase intention.

### Perceived Trust and Purchase Intention

Trust is a multi-dimensional concept, which includes cognitive, parental, and behavioral dimensions (Lewis and Weigert, 1985). Gefen et al. (2003) state that in the e-commerce environment, the concept of trust has obvious diversity. The existing research regards trust as trusting beliefs (Alba and Hutchinson, 1987; Allsop et al., 2007) and trusting intentions (Anand et al., 1988; Alreck and Settle, 1995). In e-commerce, trust is also seen as honesty. Chu and Lu (2007) define purchase intention as the degree to which consumers would desire to purchase products in the future. In this study, purchase intention refers to the extent to which customers would want to purchase products in the future after exposure to UGC advertising. The influence of trust on purchasing intention has been widely recognized in academia (Yun, 2011; Bulut and Karabulut, 2018). Trust reduces transaction risk for consumers, and plays a key role in interaction between consumers and UGC (Wirtz and Lwin, 2009; Cheng et al., 2019).

On social media, consumers cannot communicate face to face, which improves their perception of risk. Currently, trust is a basis of consumer decision-making to a large extent (Chong, 2013; Zhou et al., 2018). Today’s online consumers believe that UGC is more trustworthy than content provided by sellers (Jonas, 2010). From the perspective of economics, consumers’ purchase intention is based on the reliability of products and UGC (Ert et al., 2016). Consumers’ recommendation can influence product choice through trust (Smith et al., 2005; Bahtar and Muda, 2016). Consumers usually buy a product after reading all the UGC on the platform and being convinced by their content (Horst et al., 2007). The higher the frequency of community interaction, the more detailed and authentic the information, and the more likely consumers are to generate trust, thus improving their purchase intention. The high interaction quality of UGC improves consumers’ perception of product reliability, their impression of products or services, improves trust, reduces transaction risk, and enhances purchase intention (Wirtz and Lwin, 2009).

Therefore, this study proposes hypothesis 4:

*H4:* Perceived trust is positively related to consumers’ purchase intention.

### The Mediating Role of Perceived Usefulness and Perceived Trust

Social cognitive theory was first put forward by Bandura, an American psychologist, and states that behavior, human, and environmental factors are the determinants of interconnection and interaction (Bandura, 1986). According to social cognitive theory, perceived interactivity, as an environmental factor, will affect users’ behavior through human cognitive factors (Cyr et al., 2009; Lee et al., 2011). Social cognitive theory explores the interaction between individual and interactivity. Through UGC interaction, consumers’ perceived value of goods or services promote their purchase intentions (Chong, 2013; Zhou et al., 2018). To further analyze the cognitive human factors, this study refines the mediating variables (perceived usefulness and perceived trust) based on the value theory. The value theory helps us understand why and how individuals value things, how much they value, and how perception of these values affects social behavior.

Smet (2009) asserts three more intuitive values in his research on users’ participation intention in virtual community. Functional needs are satisfied by the quality and quantity of content received to fulfill their specific needs according to goal orientation. Emotive needs relate to social interaction, personal uses other than contractual agreements, and self-expression through information acquisition and dissemination based on users’ trust in others on the platform (Dholakia et al., 2004; Smet, 2009). Contextual needs relate to individual expectations and experiences like entertainment. Because this study mainly explores the mechanism of influencing online consumers’ purchase intention from the perspective of interaction, contextual needs are not included in the scope. Therefore, this study focuses on perceived usefulness and perceived trust as mediating variables. Based on the social cognition theory, many scholars assert that users’ perception of interactivity as an environmental factor has an impact on users’ cognition (Lee et al., 2011; Bahtar and Muda, 2016). Furthermore, perceived interactivity ultimately affects user loyalty and behavior intention through influencing their cognition and emotions (Cyr et al., 2009). In this study, consumers’ perception of UGC interaction quality will affect their behavior intention (purchase intention) by influencing their cognition (perceived usefulness and perceived trust).

User-generated content interaction quality has a positive impact on perceived usefulness (Muslim et al., 2014; Felix et al., 2016). Effective interaction with UGC can attract users’ attention, increase participation, and help transmit information. This is conducive to the function of information collection and transmission, can help others obtain opportunity benefits or key resources, and can improve the perception of the usefulness of products or services (Wu, 2007). Furthermore, only when new customers obtain enough information can they estimate and judge the value of products demanded by customers, to stimulate their purchase intention. In addition, high-quality interaction is the foundation of trust (Wu and Chang, 2005). Effective UGC interaction also meets consumers’ social needs, which is key to finding virtual communities with common attributes, where it is easier to establish close emotional ties and enhance their sense of trust (Müeller and Christandl, 2019). On social e-commerce platforms, every consumer cannot discuss and communicate face to face. Trust is one of the bases to determine consumers’ purchase intention, which affects consumers’ choice of products. Therefore, we propose the following hypotheses:

*H5:* Perceived usefulness plays a mediating role in the relationship between interaction quality of UGC and purchase intention.

*H6:* Perceived trust plays a mediating role in the relationship between interaction quality of UGC and purchase intention.

### The Moderating Role of Product Involvement

Product involvement refers to how consumers feel toward certain products and their personal level of relevant psychological needs, value, and other personal interests (Petty and Cacioppo, 1986). According to this difference, scholars divided consumers into low involvement and high involvement according to their product involvement Zaichkowsky, 1985). Product involvement affects the evaluation and actual purchase of a product by consumers. Product involvement is an important factor for consumer product choice (Belvedere and Goodwin, 2017). When consumers perceive usefulness and trust through UGC interaction, they are involved in the product, and will consider the uncertain consequences of the product, resulting in perceived risk (Rahman, 2017; Habib et al., 2021). The level of product involvement can change consumers’ perception of risk, affect concerns about the negative impact of the product, and then affect their purchase intention (Cui and Wu, 2016).

Consumers with high product involvement are more sensitive to the potential risks of shopping. They tend to search for more product information and compare and evaluate alternative brands. The whole decision-making process is more complex. However, consumers with low involvement will spend little time searching for information, and often make purchase decisions based on others’ experience (Owusu et al., 2016). For consumers with high product involvement, UGC is only one source of information among many that influence the final purchase decision, whereas high-quality UGC may be the sole source of information for consumers with low involvement (Tsai et al., 2015). Therefore, product involvement plays a key role in the relationship between UGC perception and purchase intention, and has a greater impact on consumers with low product involvement. Compared with high product involvement, consumers with low product involvement can improve their purchasing intention after referring to UGC recommendation. This is because through interaction with UGC, consumers will have a perception of usefulness and trust in products. At this time, low product involvement will reduce consumers’ doubts and promote consumers’ purchase intention. The effect of perceived usefulness and perceived trust on purchase intention will be improved. Therefore, this study puts forward the following hypotheses:

*H7:* Consumers’ product involvement plays a moderating role in the effect of perceived usefulness on purchase intention, such that the effect is stronger when product involvement is low.

*H8:* Consumers’ product involvement plays a moderating role in the effect of perceived trust on purchase intention, such that the effect is stronger when product involvement is low.

Based on the above analysis, we further propose a moderated mediation model. This means that product involvement moderates the indirect effect of UGC interaction quality on purchase intention *via* perceived usefulness and perceived trust.

*H9:* Consumers’ product involvement plays a moderating role in the indirect effect of interaction quality of UGC on purchase intention *via* perceived usefulness such that the indirect effect is stronger when product involvement is low rather than high.

*H10:* Consumers’ product involvement plays a moderating role in the indirect effect of interaction quality of UGC on purchase intention *via* perceived trust such that the indirect effect is stronger when product involvement is low rather than high.

**FIGURE 1:**
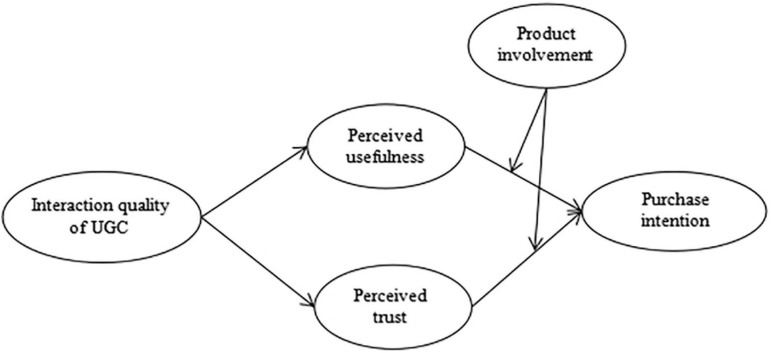
Conceptual model.

Figure 1 displays the theoretical model in the present study.

## Materials and Methods

### Sampling Procedure

To enhance the reliability of the research results, this study posted our questionnaires Sojump (http://www.sojump.com), a larg-scale online survey platform in China that is widely used in behavioral and psychological research (Li et al., 2018). SPSS 25.0 and Amos 24.0 were used for data analysis. To be included, a consumer had to have (1) used social media and (2) browsed UGC. We included the two exclusion criteria in the questionnaire at the same time, and used them to eliminate the non-conforming questionnaire when collecting the results. Before completing the questionnaire we asked participants to recall their most recent time they browsed UGC on social media to buy a product. In addition, we gave the concept of UGC and corresponding examples at the beginning of the questionnaire to ensure the quality and accuracy of the results. We obtained 280 questionnaires, and after removing those with invalid answers were left with 272 copies of effective questionnaires. The sample size meets Hair et al. (1998) recommendation. Therefore, the recovery rate was 97.14%.

The gender ratio of the sample was relatively balanced, with 57.7% males and 42.3% females. The majority had a bachelor’s degree or above, accounting for 70%. These had a good understanding of the questionnaire and were usually able to make better decisions to ensure the accuracy of the data (Hassanein and Head, 2007). Most were aged 19–30 years, accounting for 77.6%, which is also the age range of China Mobile Internet users. From the perspective of average monthly income, the overall distribution was relatively even; 62.9% had an income over 4000 RMB, which is basically consistent with the respondents’ occupation and living conditions. We can see from the sample that it is representative, which shows that the results of this study have a practical significance.

### Measures

We measured the variables in this study using established scales adapted to the context of the study. Each questionnaire item corresponding to the constructs was measured using a five-point Likert scale, anchored on “1-strongly disagree” and “5-strongly agree.” The three-item measurement of perceived interaction quality was based on the work of Ohanian (1970). The perceived usefulness of UGC was measured with three items on a five-point interval scale, previously used by Gruen et al. (2006). The three-item measurement of perceived trust was based on the work of Kim et al. (2008). Referring to the measurement scale of Misra and Beatty (1990), this study used five items to measure purchase intention. The measurement of product involvement was mainly based on the research of Zaichkowsky (1985). See Table 1 for specific items.

**TABLE 1:** Constructs, scale items and descriptive statistics.

**Variable**	**Item**	**Factor loading**	**AVE**	**CR**	**Cronbach’s α**
Interaction quality	The UGC I browse has a good interaction with me	0.701	0.506	0.752	0.741
	The UGC I browse gives me a sense of communicating product information with others	0.803			
	The UGC I browse can interest me	0.618			
Perceived usefulness	The UGC I browse is easily accessible	0.573	0.554	0.784	0.752
	The UGC I browse adds effectiveness	0.836			
	The UGC I browse adds productivity	0.796			
Perceived trust	I think the UGC’s statement is correct	0.661	0.507	0.754	0.745
	I think the UGC’s statement is dependable	0.678			
	I think the UGC’s statement is honest	0.790			
Purchase intention	After searching and browsing UGC, I have a great possibility to consider buying recommended products	0.690	0.534	0.774	0.770
	I am willing to buy products recommended in UGC	0.774			
	I’ll recommend to others the products recommended in the UGC	0.726			
Product involvement	I think it’s important for me to use this product	0.762	0.547	0.783	0.783
	I think the use of this product is closely related to my life	0.698			
	I think it’s very meaningful for me to use this product	0.757			

### Control Variables

We chose respondent gender, age, education, career, income, and purchase frequency as control variables. Age and gender have been proven to make a difference to consumer trust toward the seller (Chen et al., 2014). Mittal and Kamakura (2001) pointed out that age and gender are factors that affect consumer satisfaction and loyalty, which then affects consumer trust. In addition, purchase frequency has been proven to play an important role in online shopping behavior (O’Cass and Fenech, 2003), because consumers can develop knowledge and skills through the Internet (O’Cass and Carlson, 2012).

## Results

### Reliability and Validity

To ensure construct validity for each indicator, we conducted principal component factor analysis followed by calculation of average variance (AVE) and CR to assess the convergent validity of the measurement model. Table 1 presents the results. Data shows the outer loading value of convergent validity of each indicator exceeded the accepted value of 0.5 (Hair et al., 2013). The AVE value of all constructs exceeded the 0.5 recommended by Fornell and Larcker (1981), indicating that the scale had good convergence validity. All items had a composite reliability value above the benchmark of 0.60, indicating that the measurement model had good internal consistency. The results show that the construct of this study has adequate convergence validity. Furthermore, we also examined discriminant validity using the value of square roots of AVE, as shown in Table 2. The square root of AVE values of all latent variables was greater than the correlation coefficient with other latent variables, indicating that the discriminant validity of the scale was good (Barclay et al., 1995). The reliability of the measurement items was measured with Cronbach’s Alpha coefficient, and for all factors the values were greater than 0.7 and accepted based on George and Mallery’s (2003) criterion. These results provide a basis for the further demonstration of the hypotheses.

**TABLE 2:** Descriptive statistics correlation coefficients and discriminant validity model.

**Variable**	**1**	**2**	**3**	**4**	**5**
1 Interaction quality	0.711	0.664**	0.616**	0.643**	0.623**
2 Perceived usefulness	0.664**	0.744	0.619**	0.598**	0.520**
3 Purchase intention	0.616**	0.619**	0.731	0.655**	0.590**
4 Perceived trust	0.643**	0.598**	0.655**	0.712	0.574**
5 Product involvement	0.623**	0.520**	0.590**	0.574**	0.740
Mean	1.993	1.864	2.056	1.974	2.028
SD	0.687	0.597	0.726	0.641	0.676

*N* = 272.

*p < 0.05, **p < 0.01, and ***p < 0.001.

*The value on the diagonal represents the square root of the AVE value.*

### Hypothesis Testing

We used Amos 24.0 to construct a structural equation model to test path coefficients and the hypotheses. Table 3 shows the fitting degree between the model and the actual data. According to the existing thresholds (Fornell and Larcker, 1981; Marsh et al., 1988; Kline, 2011), the theoretical model exhibited adequate fit to the empirical data.

**TABLE 3:** Comparison of the theoretical model fit index results with the evaluation standard.

	**χ2/df**	**RMSEA**	**GFI**	**AGFI**	**IFI**	**TLI**	**CFI**	**NFI**
Evaluation Standard	<3	<0.080	>0.900	>0.800	>0.900	>0.900	>0.900	>0.900
Values in this study	2.241	0.068	0.922	0.883	0.946	0.929	0.946	0.907

**FIGURE 2:**
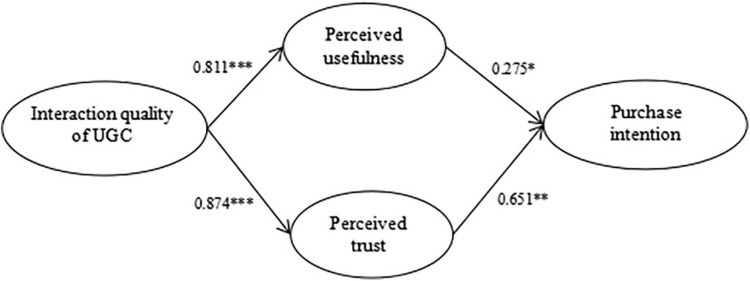
Path coefficients of the hypothesized model.

According to the test results of each standardized path coefficient in the model (Figure 2), we see that the interaction quality has a significant positive impact on perceived usefulness and perceived trust (β = 0.811, *p* < 0.001; β = 0.874, *p* < 0.001). This adequately supports Hypothesis 1 and Hypothesis 2. Meanwhile, perceived usefulness and perceived trust also have a significant positive impact on purchase intention (β = 0.275, *p* < 0.05; β = 0.651, *p* < 0.01), which verifies Hypothesis 3 and Hypothesis 4.

To examine such a mediation effect, we adopted the bootstrap method proposed by Hayes (2013). Table 4 shows the test results. LLCI and ULCI refer to lower level and upper level confidence interval, respectively. The results show that the mediating effect of perceived usefulness and perceived trust on the relationship between interaction quality and purchase intention was significant. The 95% CI did not contain 0, indicating that the mediating effect was significant. This supports Hypothesis 5 and Hypothesis 6.

**TABLE 4:** Mediating effect test.

**Effect**	**Estimated Value**	***P***	**Standard Error**	**LLCI**	**ULCI**
Interaction quality → Perceived usefulness→ Purchase intention	0.269	0.035	0.133	0.072	0.502
Interaction quality→ Perceived trust→ Purchase intention	0.686	0.000	0.160	0.473	1.002

**FIGURE 3:**
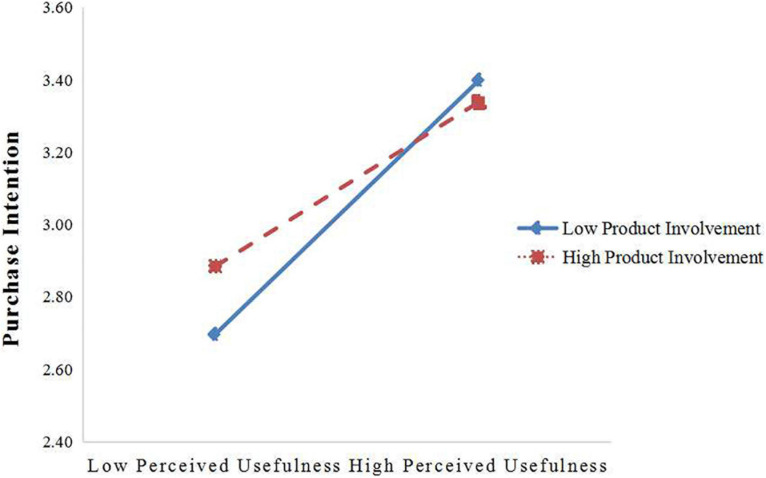
Interaction of perceived usefulness and product involvement on purchase intention.

**FIGURE 4:**
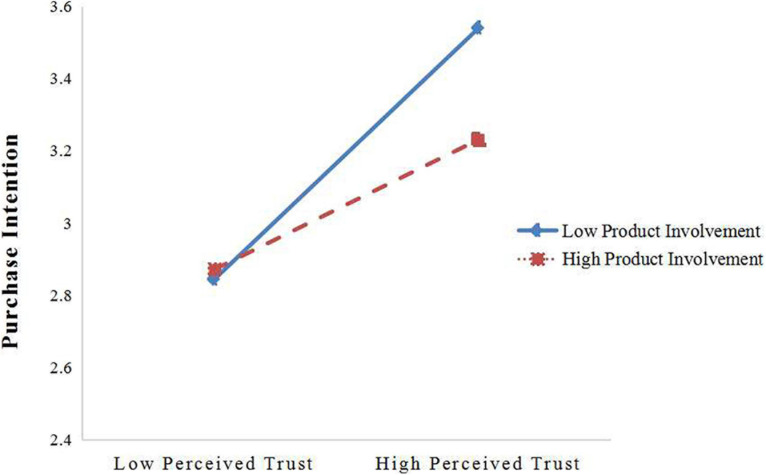
Interaction of perceived trust and product involvement on purchase intention.

To further test our moderating effect, we conducted a series of hierarchical regression analyses using SPSS 25.0 (Hox, 2010). Due to the need to verify the interaction between independent and moderating variables, to reduce the multicollinearity between variables in the regression equation, this study first centralized all variables and constructed the interaction term between them (Aiken and West, 1991). Table 5 shows the regression results. In model 4, the interaction items of perceived usefulness and product involvement had significant negative effects on consumers’ purchase intention (β = −0.095, *p* < 0.05). In model 7, the interaction items of perceived trust and product involvement had a significant negative influence on consumers’ purchasing intention (β = −0.160, *p* < 0.001). We examined the interaction effects at different levels of perceived usefulness (the mean of perceive usefulness ± 1 SD) and perceived trust (the mean of perceive trust ± 1 SD) for testing the moderation effect clearly (Aiken and West, 1991) with 95% confidence intervals and 1,000-fold bootstrap replicates for confidence intervals (Figures 3, 4). The results indicated that the relationship between perceived usefulness and purchase intention is stronger when product involvement is low (β = 0.634, *p* < 0.001) rather than high (β = 0.390, *p* < 0.001; Figure 3). Thus, the data supports Hypothesis 7. Furthermore, the relationship between perceived trust and purchase intention is stronger when product involvement is low (β = 0.720, *p* < 0.001) rather than high (β = 0.366, *p* < 0.001; Figure 4). Thus, Hypothesis 8 was supported.

**TABLE 5:** Results of the moderated regression analyses.

**Variables**	**Purchase intention**						
	**Model 1**	**Model 2**	**Model 3**	**Model 4**	**Model 5**	**Model 6**	**Model 7**
Age	−0.040	−0.041	−0.055	−0.052	−0.025	−0.041	−0.036
Gender	0.011	−0.029	−0.012	−0.011	−0.012	−0.001	0.010
Education	0.107	0.062	0.047	0.038	0.084	0.066	0.047
Occupation	0.004	−0.045	−0.045	−0.041	−0.016	−0.022	−0.023
Income	−0.044	0.004	0.029	0.024	−0.012	0.012	−0.001
Purchase frequency	−0.332***	−0.172***	−0.114*	−0.117*	−0.164***	−0.119*	−0.106*
Perceived usefulness		0.573***	0.412***	0.401***			
Perceived trust					0.608***	0.453***	0.455***
Product involvement			0.344***	0.348***		0.295***	0.322***
Perceived usefulness × Product involvement				−0.095*			
Perceived trust × Product involvement							−0.160***
*R*^2^	0.121	0.416	0.498	0.507	0.458	0.514	0.538
*ΔR*^2^	0.121***	0.296***	0.082***	0.009*	0.338***	0.055***	0.024***
*F*	6.056	133.805	43.066	4.713	164.737	29.934	13.740

*N* = 272.

*p < 0.05, **p < 0.01, and ***p < 0.001.

*Perceived usefulness, perceived trust, product involvement and the interactions were centered prior to analysis.*

Based on Hayes’ (2013) method, the last step to test the moderated mediation model is demonstrating why this mediating effect will be strengthened, weakened, or changed when the level of moderating variable is higher or lower. Thus, we designed high, medium, and low levels of product involvement (Tables 6, 7). There was a positive effect of interaction quality on purchase intention through perceived usefulness and perceived trust. However, the size was different when across levels of product involvement. Table 6 shows the conditional indirect effect of interaction quality on purchase intention through perceived usefulness was strong when the moderating variable subtracts one SD to the mean of product involvement [β = 0.28, 95% CI: (0.16, 0.41)]. The indirect effect was weak when the moderating variable adds one SD from the mean of product involvement [β = 0.17, 95% CI: (0.06, 0.28)]. Thus, Hypothesis 9 was supported. Table 7 shows the conditional indirect effect of interaction quality on purchase intention through perceived trust was strong when the moderating variable subtracts one SD to the mean of product involvement [β = 0.36, 95% CI: (0.26, 0.49)], while the indirect effect was weak when the moderating variable adds one SD from the mean of product involvement [β = 0.15, 95% CI: (0.05, 0.26)]. Thus, Hypothesis 10 was supported.

**TABLE 6:** Conditional indirect effects of interaction quality on purchase intention *via* perceived usefulness at levels of product involvement.

	**Effect**	**SE**	**Boot LLCI**	**Boot ULCI**
Low product involvement, IQ-PU-PI	0.28	0.06	0.16	0.41
Medium product involvement, IQ-PU-PI	0.23	0.05	0.14	0.33
High product involvement, IQ-PU-PI	0.17	0.06	0.06	0.28

**N* = 272.*

*IQ, Interaction Quality; PU, Perceived Usefulness; PI, Purchase Intention.*

**TABLE 7:** Conditional indirect effects of interaction quality on purchase intention *via* perceived trust at levels of product involvement.

	**Effect**	**SE**	**Boot LLCI**	**Boot ULCI**
Low product involvement, IQ-PT-PI	0.36	0.06	0.26	0.49
Medium product involvement, IQ-PT-PI	0.26	0.05	0.17	0.35
High product involvement, IQ-PT-PI	0.15	0.05	0.05	0.26

**N* = 272.*

*IQ, Interaction Quality; PT, Perceived Trust; PI, Purchase Intention.*

## Discussion

Social media has profoundly changed our communication mode and affected our purchase decisions. Even if advertisers invest more into communicating through online social networks (Chi, 2011), today’s consumers are more dependent on UGC than on product advertising (Mir and Rehman, 2013). With this view in mind, based on social cognition theory and value theory, this study proposes a research model for the impact of UGC’s interaction quality on consumers’ purchase intention. Our findings confirmed that UGC interaction quality is a critical factor in predicting consumers’ purchase intention. Specifically, our results consistently showed that UGC interaction quality had a direct relationship with consumers’ purchase intention. Both perceived usefulness and perceived trust mediate the relationship between interaction quality and purchase intention. Further, for consumers with low-level product involvement, the relationship between perceived usefulness and purchase intention is stronger as compared to high-level product involvement, as does product involvement between perceived trust and purchase intention.

### Theoretical Implication

Our research contributes to the literature in the following ways. First, based on the theory of social cognition, this study views interactivity as a key feature of Web 2.0 technology. It can help empirically analyze consumers’ cognition of UGC interaction, addressing the limitations of previous studies that only consider the information characteristics and the role of users’ social factors. It further expands the scope of antecedents affecting purchase intention, and then provides an important theoretical reference for future research in the field of UGC.

Second, to better understand how purchase intention is influenced by UGC’s interaction quality, this study views perceived usefulness and perceived trust as mediating variables based on the value theory and the coexistence of media and social media platforms. This further enriches the theoretical results of UGC behavior research on social media platforms.

Third, for information with the same content expression and dissemination direction, receivers’ understanding may differ. By studying the impact of UGC on consumers’ purchase intention in the context of social e-commerce, this study takes the characteristics of UGC information receivers into account. It introduces product involvement as a moderating variable, which provides more theoretical basis for social e-commerce to better play the role of UGC.

### Practical Implications

The current research also offers practical implications. Given the popularity of content marketing, product information created by consumers on social networks has a great impact on companies and brands. Consumer empowerment has therefore affected brand management beyond what can be controlled by companies (Arnhold, 2010). Thus, it will be useful for them to identify how UGC affects purchase intention, and this thought created the starting point of this study. Our conclusions suggest that both multi-channel retailers and other businesses cannot afford to ignore UGC as a marketing tool.

First, our research shows that UGC interaction quality is important to promote consumers’ purchase intention. Therefore, marketers should appreciate the importance of UGC, good interaction between UGC and consumers, and be committed to creating a positive experience for them. UGC can therefore be used by companies for brand-consumer interaction, which can help with customer acquisition and retention (Burmann, 2010). UGC publishers can remove single text discussion and improve interaction by using more advanced forms of audio, video, pictures, and other resources. Communication between members will be more intuitive, information will be richer and more reliable, and emotion will be more straightforward. In addition, members are encouraged to interact more frequently by means of membership levels and bonus points. This will transform weak online links into strong ones and further strengthen the marketing value of UGC platforms.

Second, to improve consumer perception of UGC and form purchase intentions, perceived usefulness and perceived trust may play a role. Based on the social cognitive theory, this study concludes that UGC interaction quality will affect consumers’ purchase intention through perceived usefulness and perceived trust. High quality interaction shows consumers that they can quickly update information, get real-time information, and actively control information acquisition on the platform (Bao et al., 2016). These features significantly improve users’ perception of usefulness. At the same time, the interactive atmosphere of social e-commerce platforms is the basis of online word-of-mouth communication. Consumers can obtain information through others’ recommendation, to reduce perceived risk and improve perceived trust. As a basis of consumer decision-making, usefulness and trust will further improve consumers’ purchase intention. Therefore, marketers should pay attention to consumers’ perception of usefulness and trust while improving interaction. In UGC interaction, more detailed information of products or services are available to give consumers the feeling of active control. This can increase the contact between consumers and UGC publishers and make them feel more closely connected. Meanwhile, two-way communication and synchronous interaction can make consumers feel that UGC publishers are approachable and willing to listen to the voice of buyers (Yoon et al., 2008). Through proper interaction, UGC publishers can demonstrate their knowledge to consumers, thereby demonstrating their ability to provide satisfactory products.

Finally, this study shows that consumers’ product involvement plays a moderating role in the indirect effect of UGC interaction quality on purchase intention *via* perceived usefulness. The indirect effect is stronger when product involvement is low rather than high, and product involvement indirectly affects UGC interaction quality on purchase intention *via* perceived trust. Therefore, we should adopt different marketing methods for products with different involvement levels. When consumers’ product involvement is low, perceived usefulness and perceived trust have a strong impact on consumers’ purchase intention. Therefore, enterprises operating low involvement products should actively use UGC’s interactivity for content marketing, which can stimulate consumers’ purchase intention. For products with high participation, consumers’ perceived risk is strong, and there are many factors to consider in making purchase decisions. Marketers need to take some measures to improve consumers’ purchase intention. They can establish their own interactive forums on social media, fully present and explain product information and value, and appropriately invite third-party professionals or professional UGC publishers to conduct a comprehensive evaluation of products, to reduce consumers’ perceived risk and improve consumers’ purchase intention.

### Limitation and Future Research

We focused on solving possible theoretical and methodological problems. However, there are still some limitations.

First, based on the survey, this cross-sectional data cannot adequately test the actual relationship between variables, and it is also difficult to show the dynamic impact between variables. Research on actual consumer behavior in UGC is rare. Future follow-up research could be more longitudinal and improve the relationship between variables. In addition, qualitative research on UGC is welcome, because this can reveal realistic behaviors related to consumer cognition and behavior.

Second, our sample only covers a specific user group in China. Whether the findings can be applied to other environments should be studied further. Future studies could use cross-cultural approaches to study the impact mechanism between these variables.

Third, the value perception of platform technology also plays an important role in promoting user behavior (Chen and Yen, 2005; Park and Chung, 2011). In this study, we did not consider whether the gradually rich level authentication mechanism and identity token as an external reward will positively affect the UGC in some social media. Later studies should consider these factors in the model to expand the scope and depth of UGC research.

In conclusion, UGC seems to be recognized as the most important driver in the future of the market (Thoumrungroje, 2014). Given the strong influence of interaction characteristic of social media, it is significant that scholars and managers develop a better understanding of how UGC interaction quality influences consumers’ cognition and behavior. We hope that the impact of UGC on consumers’ cognition and behavior is still a fruitful research topic.

## Data Availability Statement

The datasets generated for this study are available on request to the corresponding author.

## Author Contributions

RG and JC contributed to conception and design of the study. JC performed the statistical analysis. RG wrote the first draft of the manuscript. Both authors contributed to manuscript revision, read, and approved the submitted version.

## Conflict of Interest

The authors declare that the research was conducted in the absence of any commercial or financial relationships that could be construed as a potential conflict of interest.
